# Growth rate, diameter at rupture, and associated patient characteristics of abdominal aortic aneurysms without an intraluminal thrombus

**DOI:** 10.1016/j.jvssci.2026.100430

**Published:** 2026-07-14

**Authors:** Moritz Lindquist Liljeqvist, Asta Joshi, Antti Siika, Marko Bogdanovic, Patricia Andersson, Stefan Stoisavljevic, Rebecka Hultgren, Joy Roy

**Affiliations:** a Karolinska Institutet, Stockholm, Sweden; b Department of Vascular Surgery, Karolinska University Hospital, Stockholm, Sweden

**Keywords:** Aortic aneurysm, Abdominal, Intraluminal thrombus, Computed tomography, Growth rate

## Abstract

**Objective:**

The aim of this study was to provide clinical and prognostic data on the small share of abdominal aortic aneurysms (AAAs) devoid of an intraluminal thrombus (ILT).

**Methods:**

Three retrospective samples were studied: electively treated patients (n = 234, growth rates available for n = 158 and an early baseline computed tomography [CT] available for n = 128), patients with rupture (n = 133), and those followed in surveillance (n = 161). Growth rates of electively treated AAAs were calculated based on all measurements preceding surgery. ILT thickness was measured perpendicularly to the aneurysms’ centerline and was defined as *absent* if no intraluminal contrast deficit was detected, *minimal* if the ILT thickness was <5 mm, and *present* when ≥5 mm.

**Results:**

ILT was absent and minimal in 4.8% and 3.2%, respectively, in the preoperative CT of the electively treated AAAs. Patients with absent/minimal ILT appeared to suffer less cardiovascular burden, but there were no differences in anticoagulation. Absent/minimal ILT was associated with a smaller AAA diameter at baseline (*P* = 1.6e-5) and at the last preoperative CT (*P* = .0048). There were however no associations between absent/minimal ILT and growth after a baseline CT (n = 128; *P* = .67) or preceding a preoperative CT (n = 158; *P* = .091; *P* adjusted for diameter = 0.27). There were similar shares of absent/minimal ILTs in the elective and ruptured AAA groups (8% and 9%, respectively, *P* = .66). AAAs with absent/minimal ILTs ruptured at smaller aneurysm diameters (median, 68 vs 79 mm; *P* = .036) and absent/minimal ILT was associated with rupture adjusted for diameter (*P* = .037) but not when also adjusting for other patient characteristics (*P* = .89). Similar results were observed in the cohort of patients with AAAs under surveillance.

**Conclusions:**

The absence of ILT is more common in smaller AAAs but is not associated with growth rate. AAAs without ILT seem to rupture at smaller diameters, but ILT absence is not an independent rupture risk factor.

**Clinical Relevance:**

The present study analyzed patients with abdominal aortic aneurysms (AAA) that do not develop an intraluminal thrombus (ILT). It was shown that the absence of ILT was associated with smaller maximal diameters in asymptomatic and ruptured AAAs, but there was no difference in growth rate between the groups, and the absence of ILT was not an independent risk factor for rupture. Collectively, these data did not support specific management considerations in patients with AAAs without an ILT.

**Article Highlights**

• **Type of Research:** This was a single-center, case-control observational study
• **Key Findings:** This study analyzed the scarcely described small share of patients with an abdominal aortic aneurysm (AAA) that does not contain an intraluminal thrombus (ILT). It was found that patients with an ILT-free AAA tended to have less atherosclerotic burden but were otherwise similar to those with an ILT-containing AAA. The absence or, if present, thickness of the ILT was not associated with AAA growth rate. While ILT-free AAAs ruptured at smaller diameters than those with an ILT, ILT absence was not an independent rupture risk factor.
• **Take Home Message:** The study did not support specific management considerations of patients with an ILT-free AAA.

Abdominal aortic aneurysm (AAA) is the irreversible dilatation of the abdominal aorta, in clinical practice defined as reaching a diameter larger than 30 mm.^1^ In AAAs of the common, degenerative etiology, this dilatation results from the progressive degradation of normal vascular extracellular matrix with loss of elastin and smooth muscle cells and a gain of irregular fibrosis.^2^^,^^3^ If the AAA is left untreated, there is a risk of rupture, a lethal condition from which most patients succumb, and a 40% mortality rate for those who survive long enough to reach vascular surgery.^4^^,^^5^ There is no medical treatment for AAA, and in order to prevent rupture, endovascular or open aneurysm repair is performed.^6^^,^^7^ However, elective aortic surgery carries a risk of morbidity and mortality, and the risk of rupture must be weighed against the operative risks. The risk of rupture has been found to increase with a larger AAA diameter.^8^ Four randomized controlled trials have evaluated surgery for AAAs with a diameter smaller than 55 mm without finding an effect on mortality.^9^ However, there is a remaining risk of rupture in small AAAs under surveillance, and factors such as female sex, smoking, the geometry and estimated biomechanical state of the AAA, have been identified as rupture risk predictors, and their use has been proposed to individualize AAA management.^7^^,^^10^, ^11^, ^12^ An active area of research directed at finding predictors of growth and rupture concerns the intraluminal thrombus (ILT) that most AAAs contain.^1^^,^^13^

A chronic, nonocclusive ILT typically forms in AAAs over time and is apparent on contrast-enhanced computed tomography (CT) as an intraluminal contrast deficit. Several efforts have been made to map the potential role of ILT in AAA growth and rupture, with sometimes seemingly contradictory results.^14^ Its formation may be linked to a disruption of aortic endothelium as well as altered and prothrombotic hemodynamics of the enlarged abdominal aorta.^14^, ^15^, ^16^ Once formed, the ILT takes on a multilayered structure with erythrocytes and leukocytes trapped within the fibrin deposition. There is an aggregation of active leukocytes and proteases within the ILT, and the underlying vessel wall exhibits increased inflammation, matrix degradation, smooth muscle cell loss, and decreased biomechanical strength, seemingly contributing to aneurysm rupture.^2^^,^^17^, ^18^, ^19^ However, the ILT has been found to cushion the vessel wall against biomechanical stress.^20^

While most AAAs develop an ILT over time, a minority do not.^13^ There are scarce data regarding the prognosis and characteristics of AAAs without an ILT. Analyzing this subset could help individualize AAA management and shed light on the role of the ILT.

The aim of the current study was to compare patient characteristics, growth rate, and diameter at rupture between AAAs with and without an ILT.

## Methods

### Patients—Elective cohort

Of 281 patients with AAA scheduled for open or endovascular repair of an AAA at the Karolinska University Hospital between 2016 and 2020 and who had been consecutively enrolled to participate in the Stockholm AAA Biobank,^19^ 234 were included after informed consent was collected. There was no patient overlap with the cited publication. Inclusion criteria were a diagnosis of AAA, planned elective surgery of the same, and at least one contrast-enhanced CT examination. Exclusion criteria were inability to provide informed consent; aortic dissection as the primary diagnosis as well as aortic infection; vasculitis; rupture; an aneurysm secondary to known genetic connective tissue disorders or administrative causes, such as inaccessible medical records or images.

### Patients—Rupture cohort

A previously published cohort comprising all 283 patients who presented and were diagnosed with a ruptured AAA at any hospital in the Stockholm County and the island of Gotland in Sweden between the years 2009 to 2013 was examined.^21^ Inclusion criteria were an available contrast-enhanced CT at the time of rupture of sufficient quality to confidently determine the presence of ILT. A total of 133 patients with ruptured AAA were included in the present study.

### Patients—Surveillance cohort

A previously published cohort of patients with AAAs measuring 40 to 50 mm at baseline and who were followed for at least 4 years was used in order to ensure that the findings from the first cohorts applied to AAAs under surveillance that do not necessarily progress to surgery.^11^ Of the patients in the original cohort, n = 161 were not included in the other aspects of this study and could be included for this confirmatory analysis. The outcomes were defined as reaching an indication for surgery within 4 years or the occurrence of symptoms/rupture at any time.

### Growth rate and ILT evaluation

Diameter growth rates were calculated based on clinical measurements on CT or ultrasound (US). All AAA measurements available in the Picture Archiving and Communication System or electronic health care records, which include all evaluations performed in the larger Stockholm region from diagnosis to surgery, were used. For inclusion in the growth rate analysis, the patients’ measurement series were required to include at least two measurements of the same modality, US or CT, performed with at least an 8-month interval in order to decrease the effect of systematic diameter differences between CT and US.

Growth rates were determined by linear regression in two manners, either including all measurements or those ensuing the first CT in which the ILT could be evaluated. A separate linear regression was performed on each patient. The slope of the resulting model represented growth rate in millimeters per year. The motivation behind this approach, in contrast to the more complex nonlinear analyses used in previous literature, is recently published data showing generally continuous and linear diameter growth patterns of AAAs.^22^^,^^23^ As a sensitivity analysis, growth rate calculations were repeated using CT measurements exclusively.

Presence and thickness of an ILT were evaluated by the authors in one or, if available, two contrast-enhanced CTs per patient: the first available CT and the preoperative (last) CT. Complete absence of intraluminal contrast deficit was considered absence of ILT. Any contrast deficit was deemed to be ILT and was measured orthogonally to the AAA centerline at its thickest aspect at the level of the maximal aneurysm diameter in the Sectra IDS7 (Sectra AB) Picture Archiving and Communication System.

### Statistical analyses

For evaluation of the association between ILT thickness and diameter growth rates, Spearman’s correlation test was used. Associations between two categorical variables were evaluated with the *χ*^2^ test, and those between one categorical variable and one continuous variable with the Kruskal-Wallis test or the Wilcoxon rank-sum test. Multiple linear regression and, as a sensitivity analysis, linear mixed-effects modeling including a random intercept and slope for each subject, in order to account for subject-level variability,^24^ were performed to test associations between predictive variables, growth rates, and rupture. Associations between binary dependent and continuous or categorical independent variables were tested with logistic regression. All analyses were performed by the use of the R platform and language (R Foundation for Statistical Computing).^25^

### Ethics statement

The collection and study of patient data, radiological images, aneurysm tissue, and, in the case of ruptured AAAs, waiver of informed consent were approved by the Regional Ethical Review Board in Stockholm (00-337, 2009/9-31/4, 2016_89-31).

## Results

### Patient characteristics and ILT presence

A total of 234 asymptomatic patients were included in the elective cohort. Most asymptomatic AAAs of patients scheduled for surgery contained an ILT, with only 10 (4.3%) and 8 (3.4%) with absent or minimal ILTs, respectively. Cardiovascular disease was less prominent in patients with absent/minimal ILT, as these patients had a decreased prevalence of previous myocardial infarction and beta-blocker treatment, and none had diagnoses of angina pectoris or peripheral arterial occlusive disease (Table I). Otherwise, no differences in patient characteristics between aneurysms with present or absent/minimal ILT were found, including for anticoagulation prescriptions. For the 125 patients who had been imaged at least twice by contrast-enhanced CT, there was a significant increase in ILT presence between the first and last CT (*P* = 1.8e-8), from 74% to 92% present, 8.8% to 4.8% minimal, and 17% to 3.2% absent ILTs. All AAAs with an ILT present at the first CT retained the ILT to the second CT. However, the ILT of one patient’s aneurysm decreased from 14 to 3 mm in thickness, resulting in the ILT being classified as “present” on the first and “minimal” on the last CT.

**Table I:** Characteristics of patients with asymptomatic abdominal aortic aneurysm (AAA) in the elective cohort

	Absent/minimal (N = 18)	Present (N = 216)	*P* value
Sex (male)	13 (72%)	182 (84%)	.188
Age, years old, median (Q1, Q3)	74 (70, 79)	75 (70, 79)	.990
Family history	1 (11%)	39 (25%)	.331
Missing	9	63	
Hypertension	16 (89%)	160 (74%)	.162
Previous myocardial infarction	1 (6%)	58 (27%)	.046∗
Angina pectoris	0 (0%)	27 (13%)	.110
Missing	0	1	
Diabetes mellitus (type 2)	6 (33%)	46 (21%)	.243
Missing	0	1	
Peripheral arterial occlusive disease	0 (0%)	26 (12%)	.118
Smoking			.185
Current	2 (11%)	54 (25%)	
Previous/never	16 (89%)	162 (75%)	
Aspirin	13 (72%)	163 (75%)	.760
Clopidogrel	2 (11%)	14 (6%)	.455
Warfarin	2 (11%)	15 (7%)	.517
Missing	0	1	
Other anticoagulants including DOAC	1 (6%)	24 (11%)	.460
Missing	0	1	
ACE inhibitor	4 (22%)	69 (32%)	.392
ATII inhibitor	7 (39%)	62 (29%)	.363
Beta blocker	4 (22%)	120 (56%)	.006∗∗
Calcium channel blocker	8 (44%)	77 (36%)	.456
Statin	11 (61%)	170 (79%)	.079
Missing	0	1	

*ACE*, Angiotensin-converting enzyme; *ATII*, angiotensin II; *DOAC*, direct oral anticoagulant.

*P* values were calculated with Wilcoxon rank-sum or *χ*^2^ tests for continuous and categorical variables, respectively. ∗*P* < .05; ∗∗*P* < .005.

### Diameter growth rate, ILT presence, and patient characteristics

**Fig 1:**
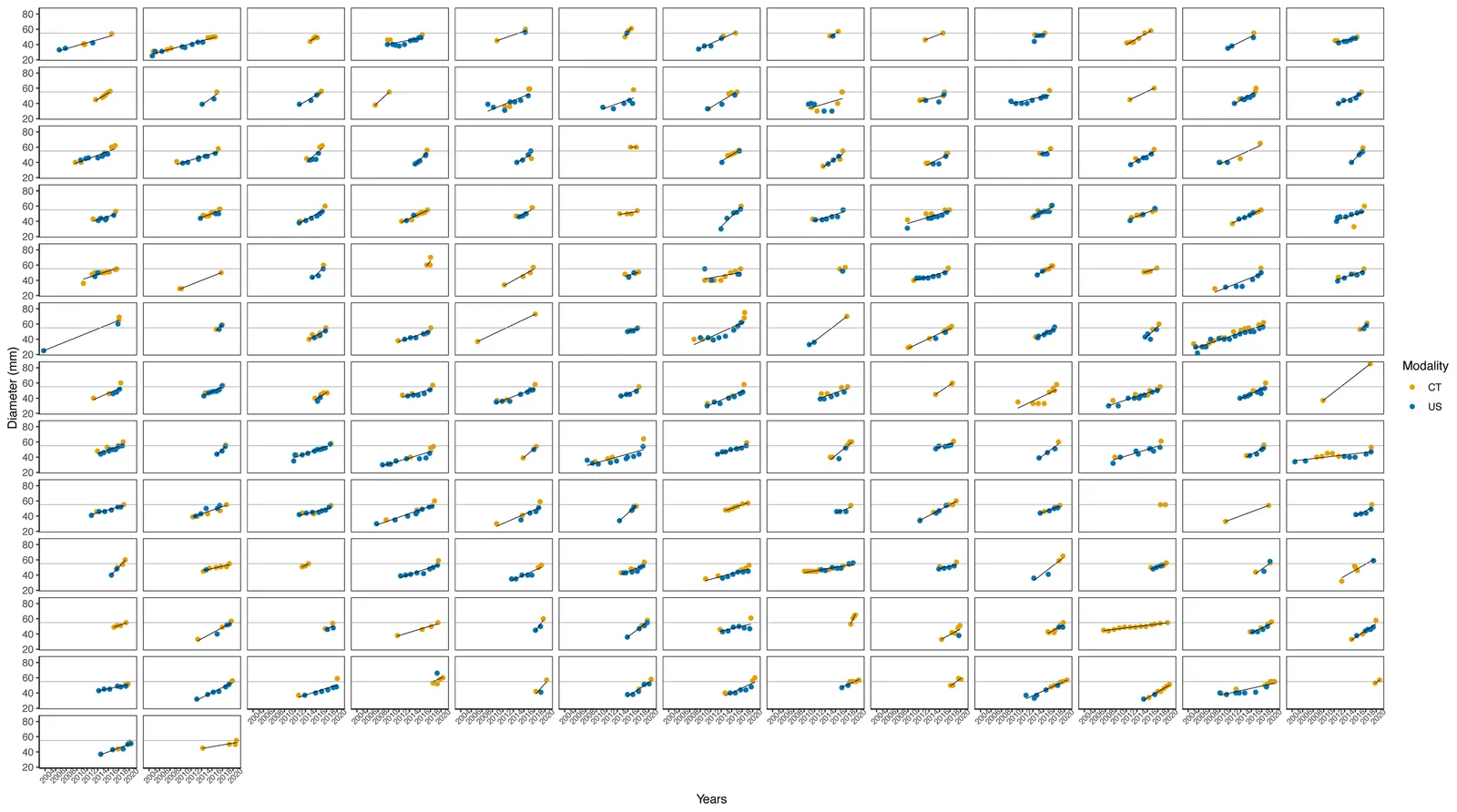
Diameter measurements over time. All measurements of included aneurysms with longitudinal data. *CT*, Computed tomography; *US*, ultrasound.

**Fig 2:**
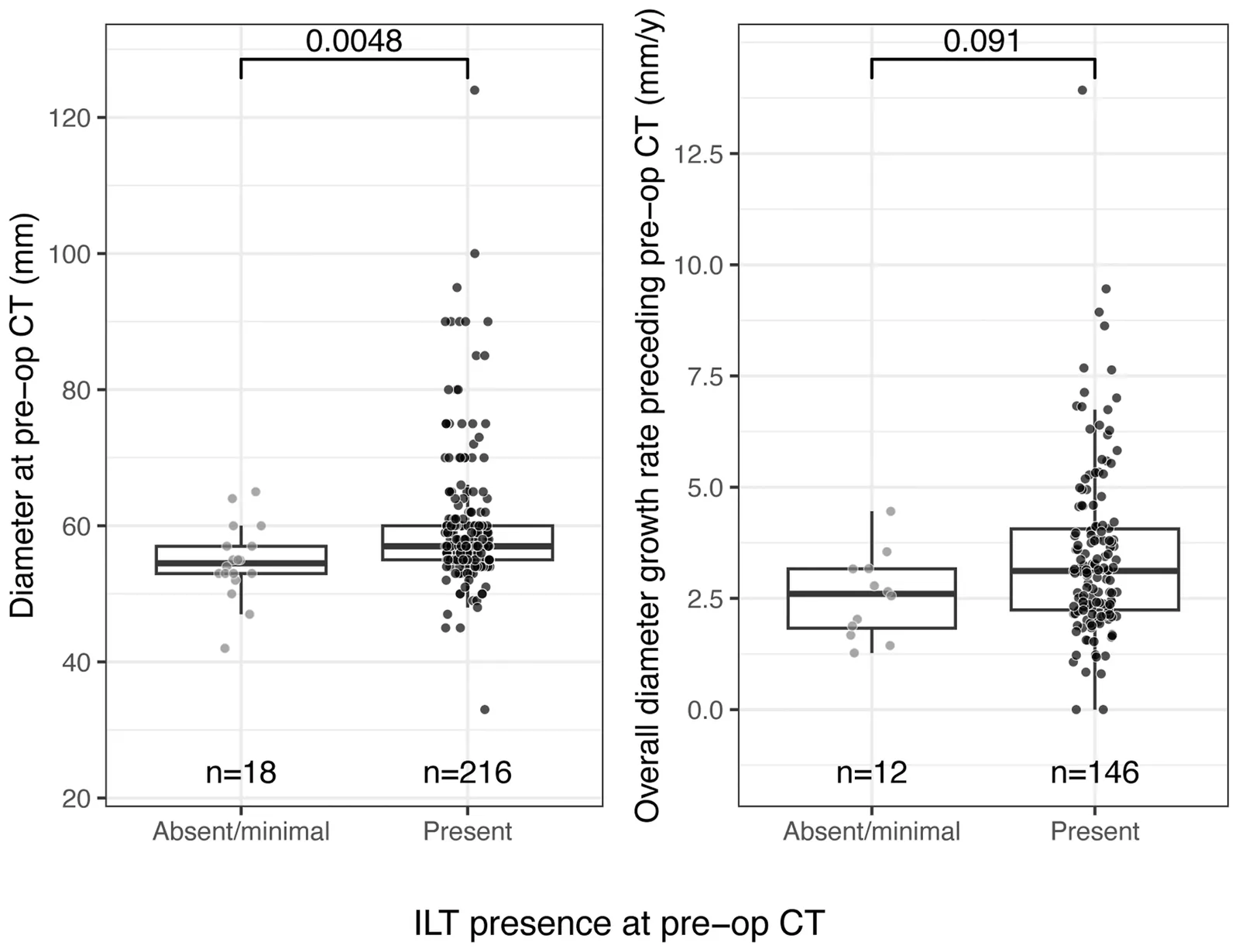
Diameter at and growth rate preceding the preoperative CT. *P* values were calculated with the Wilcoxon rank-sum test, and growth rate was determined by linear regression. *CT*, Computed tomography; *ILT*, intraluminal thrombus.

Of the 234 included patients, growth rates preceding a preoperative CT angiography could be estimated in 158 patients with a combined total of 1146 diameter measurements with CT and US over a median of 4.7 (interquartile range, 3.0-6.8) years. An overview of all measurements and growth rates is presented in Fig 1 and Supplementary Fig (online only). At the last preoperative CT, AAAs with an ILT had a larger diameter compared with those without. There was however no difference between the preceding growth rates in the aneurysms that underwent several measurements (Fig 2). This result remained in multiple linear regression with adjustment for the preoperative diameter (*P* = .27). There was no correlation between the preoperative ILT thickness and the preceding growth rate (*r* = 0.017; *P* = .82).

**Fig 3:**
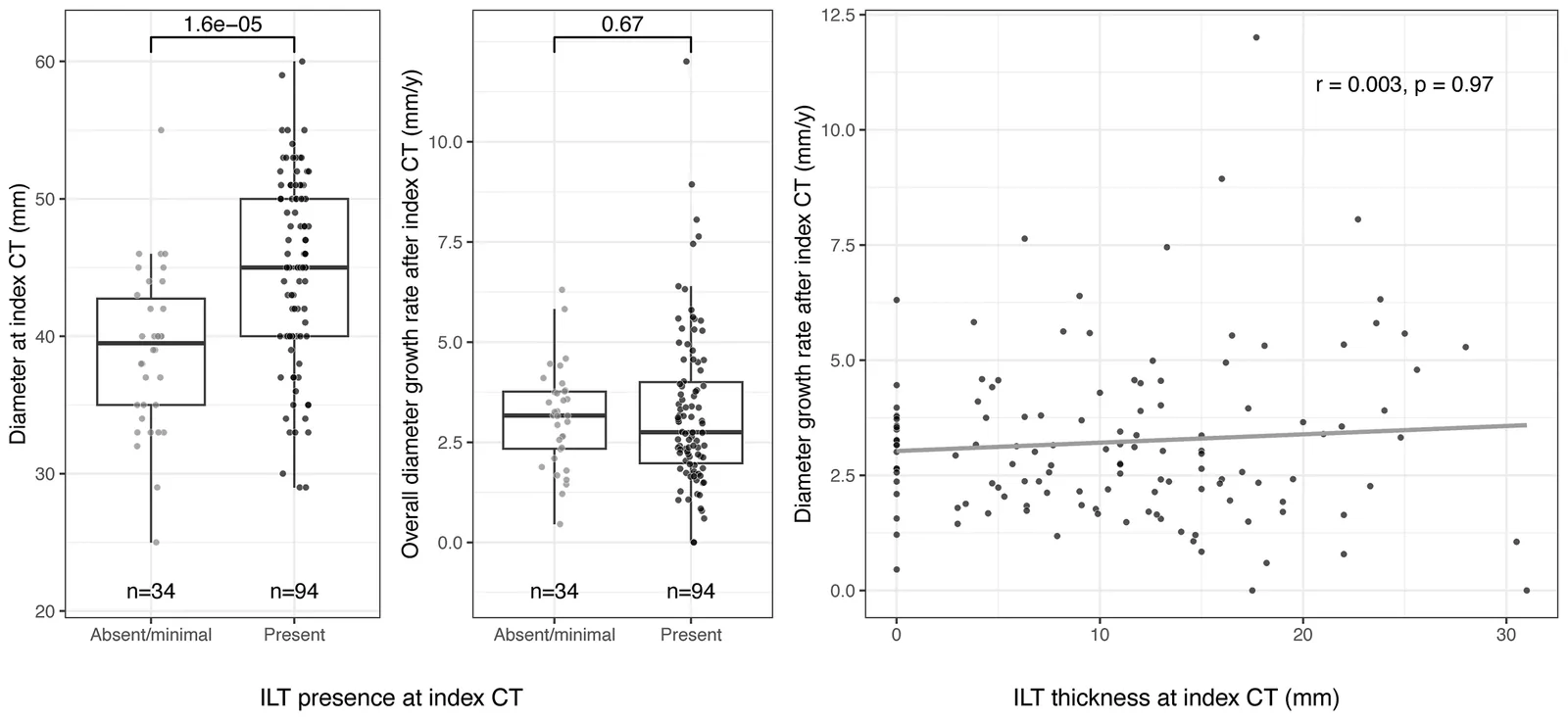
Diameter, ILT thickness, and growth rate after an index CT. *P* values were calculated with the Wilcoxon rank-sum test, and growth rate was determined by linear regression; correlation was tested with Spearman’s rank. *CT*, Computed tomography; *ILT*, intraluminal thrombus.

The ILT presence and thickness were also evaluated at the baseline CT to assess the predictive potential of these variables. Growth rate after an index CT could be studied until surgery for 128 patients. Again, the presence of ILT was associated with larger diameters, but there were no associations between ILT presence or thickness at the baseline examination with the ensuing growth rate (Fig 3). These results remained when the follow-up time after index CT was restricted to 4 or 2 years, if only CT measurements were used (ie, excluding those made by the use of US), using only baseline diameters >4 cm, in multiple linear regression, mixed-effects modeling adjusted for baseline diameter and when the three ILT categories—“absent,” “minimal,” and “present”—were analyzed separately (not shown).

Overall growth rate was negatively associated with a diagnosis of diabetes and treatment with calcium channel blockers at the time of scheduled surgery in simple linear regression (Table II). Negative effects of diabetes, calcium channel blockers, and male sex on growth rate were observed in multiple linear regression (Table III). Similar results were observed when only CT measurements were used for growth calculations and in linear mixed-effects modeling (not shown).

**Table II:** Simple linear regression of patient characteristics and aneurysm growth rate in asymptomatic patients of the elective cohort

	Estimate	*P* value
Sex (male)	−0.24	.57
Age (years old)	0	.9
Family history	−0.2	.65
Hypertension	−0.14	.69
Previous MI	−0.56	.096
Angina pectoris	−0.18	.71
Diabetes mellitus (type 2)	−0.87	.012∗
PAD	−0.48	.27
Smoking	−0.32	.34
Aspirin	0.28	.45
Clopidogrel	0.21	.69
Warfarin	−0.29	.64
Other anticoagulants including DOAC	−0.54	.27
ACE inhibitor	−0.15	.65
ATII inhibitor	0.06	.85
Beta blocker	−0.36	.22
Calcium channel blocker	−0.66	.031∗
Statin	−0.42	.25
Baseline diameter (mm)	0.01	.51

*ACE*, Angiotensin-converting enzyme; *ATII*, angiotensin II; *CT*, computed tomography; *DOAC*, direct oral anticoagulant.

∗*P* < .05.

**Table III:** Multiple linear regression of patient characteristics and aneurysm growth rate in asymptomatic patients of the elective cohort

	Estimate	value
Sex (male)	−0.98	.0047∗∗
Age (years old)	−0.02	.29
Smoking	−0.18	.54
Baseline diameter (mm)	0.03	.15
Calcium channel blocker	−0.68	.011∗
Diabetes mellitus (type 2)	−1.03	<0.001∗∗∗

∗*P* < .05; ∗∗*P* < .005; ∗∗∗*P* < .001.

### Presence of ILT in ruptured AAAs

**Fig 4:**
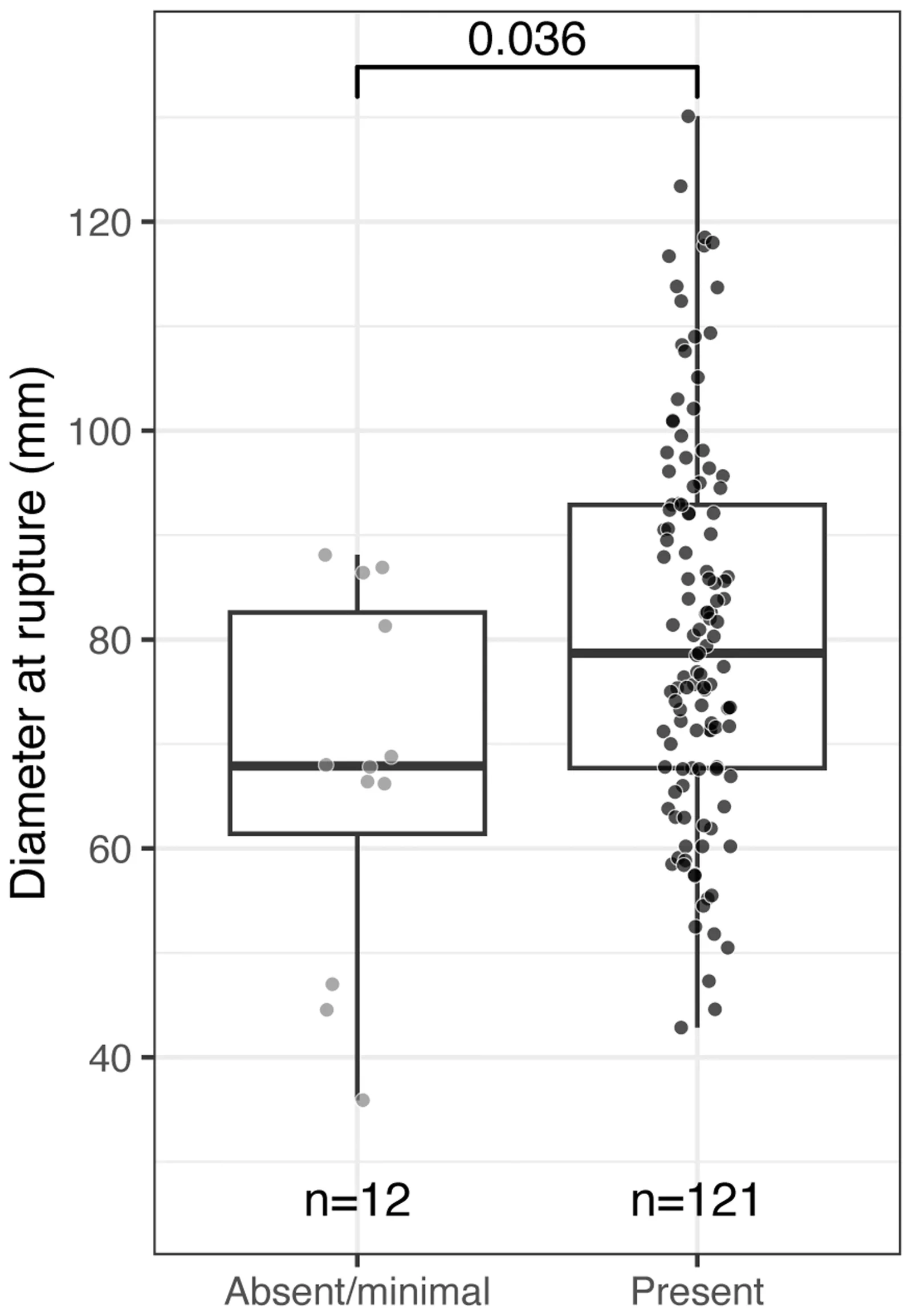
Diameter at rupture. *P* value was calculated with Wilcoxon rank-sum test.

A total of 133 patients with a ruptured AAA imaged by a contrast-enhanced CT of sufficient quality were included. Among these patients, those with no ILT or minimal ILT suffered from rupture at smaller aneurysm diameters compared with those with an ILT present (Fig 4). There were no other significant differences in available patient characteristics (Table IV).

**Table IV:** Characteristics of patients with ruptured abdominal aortic aneurysm (AAA)

	Absent/minimal (N = 12)	Present (N = 121)	*P* value
Age, years old, median (Q1, Q3)	81 (69, 84)	79 (72, 84)	.959
Sex (female)	2 (17%)	31 (26%)	.493
Smoking			.809
Current	2 (50%)	32 (44%)	
Previous/never	2 (50%)	41 (56%)	
Missing	8	48	
Diabetes (type 2)	2 (17%)	21 (19%)	.871
Missing	0	8	
Hypertension	7 (58%)	75 (66%)	.577
Missing	0	8	

*P* values were calculated with Wilcoxon rank-sum test or *χ*^2^ test for continuous and categorical variables, respectively.

The elective and rupture groups were compared. The share of AAAs with absent/minimal ILT was similar in the two groups (elective, n = 18 [8%] and ruptured, n = 12 [9%], *P* = .66). Instead, rupture was associated with age, current smoking, aneurysm diameter, and ILT thickness (Table V). There were trends toward a higher risk in female patients and fewer diagnoses of hypertension. When adjusted for maximal diameter, there was a positive association between rupture and absent/minimal ILT (*P* = .037) but not between rupture and ILT thickness (*P* = .19). In multivariable logistic regression, positive associations between rupture and age, smoking, and aneurysm diameter remained, but absent/minimal ILT was not associated with rupture independently of these variables (Table VI).

**Table V:** Patient characteristics of the elective and ruptured samples

	Elective (N = 234)	Rupture (N = 133)	*P* value
*Age*, years old, median (Q1, Q3)	75 (70, 79)	79 (71, 84)	<.001∗∗∗
Sex (female)	39 (17%)	33 (25%)	.059
Smoking			<.001∗∗∗
Previous/never	178 (76%)	43 (56%)	
Current	56 (24%)	34 (44%)	
Missing	0	56	
Hypertension	176 (75%)	82 (66%)	.054
Missing	0	8	
Diabetes (type 2)	52 (22%)	23 (18%)	.385
Missing	1	8	
ILT			.655
Present	216 (92%)	121 (91%)	
Absent/minimal	18 (8%)	12 (9%)	
ILT thickness, mm, median (Q1, Q3)	19.7 (12.7, 25.6)	25.0 (16.0, 37.0)	<.001∗∗∗
Diameter at last CT, mm, median (Q1, Q3)	57.0 (55.0, 60.0)	77.4 (67.6, 92.1)	<.001∗∗∗

*CT*, Computed tomography; *ILT*, intraluminal thrombus.

*P* values were calculated with Wilcoxon rank-sum or *χ*^2^ tests for continuous and categorical variables, respectively. ∗∗∗*P* < .001.

**Table VI:** Multiple logistic regression of patient characteristics and risk of rupture

	Estimate	*P* value
Age (years old)	0.06	.01∗
Sex (female)	0.53	.19
Smoking (current)	1.07	.0027∗∗
Hypertension	−0.28	.45
Diabetes (type 2)	0.68	.077
ILT (absent/minimal)	0.10	.89
Diameter at last CT (mm)	0.09	<.001∗∗∗

*CT*, Computed tomography; *ILT*, intraluminal thrombus.

∗*P* < .05; ∗∗*P* < .005; ∗∗∗*P* < .001.

### Presence of ILT in a surveillance cohort

As a supportive analysis, a surveillance cohort of 161 patients was analyzed (Supplementary Table, online only). This sample was included from a previously published project^11^ and comprised patients not included in the above analyses, identified from AAA surveillance with a baseline diameter of 40 to 50 mm. A total of 78 (48%) patients reached indication for surgery within 4 years, and 11 patients (7%) became symptomatic or suffered from rupture after a median of 3 (interquartile range, 2.3-4) years. There was no significant association between ILT thickness or presence (defined as a thickness ≥5 mm) at baseline and reaching indication for surgery within 4 years, as adjusted for baseline maximal diameter in logistic regression. Similarly, there was no difference between intact/electively treated and ruptured/symptomatic AAAs with respect to ILT thickness (12 vs 15 mm, *P* = .14) or share of AAAs without ILT (15% vs 9.1%, *P* = .9).

## Discussion

The present study found no conclusive differences in patient characteristics or growth rates between AAAs with and without ILT. Among ruptured AAAs, those without an ILT were smaller, and ILT absence was a rupture risk factor when adjustment was made for diameter but not when further known rupture risk factors were included in the analysis. Similar results were observed in a separate cohort under surveillance. Collectively, these data do not support a specific treatment algorithm for AAAs devoid of ILT.

It has previously been described that most AAAs of a diameter larger than around 40 mm contain an ILT.^13^^,^^26^^,^^27^ This was also apparent in the present data, with a majority of AAAs at both index and preoperative CT containing significant thrombus. AAAs without or with minimal ILT were smaller than those with an ILT, and the prevalence of ILT increased over time with increasing average diameters. This is consistent with previous studies demonstrating growth of ILT rather than the de facto lumen when the AAAs’ external diameter expands.^23^^,^^28^

Although AAAs with an ILT were larger than those with absent or minimal ILT, there was no association between the presence or thickness of the ILT and the aneurysm’s growth rate. This lack of association held when the interval growth rate was related to the ILT at either the baseline or final (preoperative) CT, as well as when the growth rate was measured only between two CTs or if the follow-up was limited to 2 or 4 years. These results are congruent only with a share of the contrasting findings in previous literature. In some studies, faster growth rates with a larger ILT^20^^,^^28^ have been observed, whereas others found negative associations.^29^ It has further been suggested that the orientation of the thrombus deposition^30^ and magnetic resonance imaging-estimated composition^31^ could predict AAA expansion and that there may exist a U-shaped association between local thrombus thickness and local diameter growth rate.^32^ These studies were all observational and pioneering in nature, with relatively small sample sizes. A recent analysis of a sample comprising smaller aneurysms but in numbers similar to the present study found higher growth rates in ILT-containing compared with ILT-free AAAs, but this association only held for the small AAAs.^27^ Thus, as the present study included mainly larger aneurysms, our results are consistent with the cited study.

Although the absence of ILT was associated with rupture at smaller diameters, this was not an independent rupture risk factor. Previous studies investigating associations between ILT and rupture have implicated fast thrombus growth,^33^ hyperattenuating “crescent” and localized areas as apparent on CTs,^34^, ^35^, ^36^ and a larger lumen area^12^ as rupture risk factors. On the other hand, the volume of the ILT itself did not differ between ruptured and intact AAAs in a previous study.^37^ Another study found, in contrast to the herein presented results, that greater ILT thickness was associated with rupture at lower diameters.^38^ Finally, a systematic review showed that although the ILT was larger in ruptured AAAs, so was the diameter, and the studies matching for diameter found no difference in the size of the ILT.^39^ With the previous literature and the present study in mind, it is difficult to draw firm conclusions on the influence of the ILT on rupture risk, but most results to date speak against an increased risk of rupture with the presence and thickness of an ILT.

Associations between patient characteristics, growth rate, and rupture were studied in part to infer adequate power and quality of data of the present study, as well as for an exploratory purpose on the detailed long-term growth data. A negative association could be observed between diabetes and growth rate, as has been established previously in large meta-analyses,^10^ but also with the prescription of calcium channel blockers. However, calcium channel blockers have been shown to be unsuccessful in significantly reducing growth in a small randomized controlled trial with a 2-year follow-up.^40^ There was an increased share of current smoking, higher age, and a larger maximal diameter in the rupture group, as well as a trend toward a larger share of women, which correspond with previously published results.^10^

There are several limitations to the presented analyses that merit consideration. The asymptomatic patients in the electie cohort were all eventually scheduled for AAA surgery, and therefore, any patients whose aneurysms did not expand to reach the indication for surgery or, conversely, ruptured at smaller diameters were not included. To account for these limitations, we also analyzed ruptured AAAs and small AAAs under surveillance from separate cohorts, with similar results. Information on medication was only available in the asymptomatic cohorts and based on preoperative prescription data, precluding analysis of length of treatment, compliance, etc. Further, we only analyzed the presence and thickness of ILT, not type or volume, despite previous studies describing predictive value of hyperattenuations, the “crescent sign,” geometric configurations, and tissue characteristics.^30^^,^^34^, ^35^, ^36^^,^^41^ The cutoff of 5 mm for the definition of “minimal” ILT was arbitrarily chosen, and to ensure not missing results from this discretization, it was complemented by the continuous variable of ILT thickness. The focus on the presence and thickness of ILT was chosen for the present study because of its immediate clinical availability upon CT examination in any radiological software. While ILT volume is an interesting variable to study, it requires specialized software. A very strong correlation between ILT thickness and volume has been noted in previous studies.^11^ However, potential effects of ILT heterogeneity or its geometrical distribution would not be detected by the presented analyses. The use of three different cohorts could introduce heterogeneity in imaging and decision-making; however, all patients were managed from 2009 to 2020 in the same health care system, and while diameter measurements could be imprecise in rupture CT reports, the authors remeasured these diameters. Finally, the number of ruptures occurring in AAAs without an ILT was low, which limits the power of and number of covariates in analyses performed on the rupture cohort.

## Conclusions

Patients with an ILT-free AAA show a clinical tendency toward less atherosclerotic burden, but there were no convincing differences in other patient characteristics or aneurysm growth rates between patients with ILT-free or ILT-containing AAAs, and the diameter-adjusted growth rate is not associated with ILT thickness, as investigated in both a surgically treated cohort and in patients under surveillance. While the absence of ILT is associated with rupture at smaller diameters and is a risk factor for rupture adjusted for diameter, this association is not independent of other risk factors. The sample size and study design were sufficient to identify previously observed growth and rupture risk factors. Collectively, these data did not support specific management considerations in patients with AAAs devoid of ILT. Due to the scarcity of clinically significant AAAs without ILT, further investigations in larger data sets are warranted.

## Funding

This research was supported by the 10.13039/501100003793Swedish Heart Lung Foundation (grant numbers: 20230697, 20230428, and 20220451), ALF-grant from Region Stockholm, and the MedTechLab grant.

## Declaration of Generative AI and AI-Assisted Technologies in the Writing Process

Generative artificial intelligence was not used in data analysis or manuscript preparation.

## Disclosures

None.

## Figures and Tables

**Supplementary Fig (online only):**
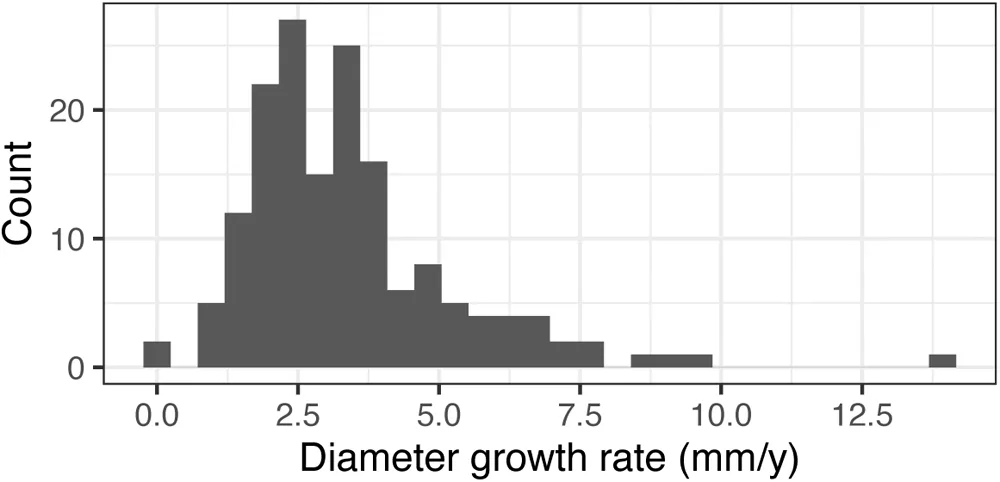
Aneurysm growth rates. Distribution of growth rates in millimeters per year preceding a preoperative computed tomography angiography in 158 patients.
