# Supplementary material for: Growth rate, diameter at rupture, and associated patient characteristics of abdominal aortic aneurysms without an intraluminal thrombus
Source: JVS Vasc Sci. 2026 Jul 14;7:100430. doi: 10.1016/j.jvssci.2026.100430 (PMC13499417; doi:10.1016/j.jvssci.2026.100430)
Supplement: Supplementary file 1 [file mmc1.docx]

**Supplemental Table. Characteristics of patients in the surveillance cohort.**

|  | **Stable**  **(N=83)** | **Surgery Indication (N=78)** | **p value** |
| --- | --- | --- | --- |
| **Patient sex** (female) | 15 (18%) | 23 (29%) | 0.088 |
| **Age** (years) | 73.12 (67.15, 78.38) | 73.20 (66.94, 78.00) | 0.930 |
| **Smoking status** |  |  | 0.602 |
| current | 24 (29%) | 27 (35%) |  |
| previous | 47 (57%) | 38 (49%) |  |
| never | 12 (14%) | 13 (17%) |  |
| **Family History of AAA** | 6 (13%) | 12 (24%) | 0.155 |
| Missing | 36 | 28 |  |
| **Diabetes** (type 2) | 10 (12%) | 8 (10%) | 0.718 |
| **Maximal diameter** (mm) | 45.4 (42.6, 47.4) | 50.4 (48.4, 52.6) | < 0.001 |
| **Max ILT thickness** (mm) | 10.8 (7.7, 15.2) | 12.8 (8.8, 18.4) | 0.036 |

Continuous values are presented as median (interquartile range) and categorical as number (percent). P-values were calculated with Wilcoxon rank-sum or chi-square tests for continuous and categorical variables, respectively.
